# Application of *Lactobaillus salivarius* WB21 to the Oral Care of Healthy Older Adults: A Randomized, Double-Blind, Placebo-Controlled Crossover Comparative Study

**DOI:** 10.3390/life12091422

**Published:** 2022-09-13

**Authors:** Satoko Kijima, Nao Suzuki, Takashi Hanioka, Masahiro Yoneda, Kazunari Tanabe, Takao Hirofuji

**Affiliations:** 1 Department of Preventive and Public Health Dentistry, Fukuoka Dental College, 2-15-1 Tamura, Sawara-ku, Fukuoka 814-0193, Japan; 2 Oral Medicine Research Center, Fukuoka Dental College, 2-15-1 Tamura, Sawara-ku, Fukuoka 814-0193, Japan; 3 Faculty of Health Care Sciences, Takarazuka University of Medical and Health Care, 1 Hanayashiki-Midorigaoka, Takarazuka 666-0162, Japan; 4 Department of General Dentistry, Fukuoka Dental College, 2-15-1 Tamura, Sawara-ku, Fukuoka 814-0193, Japan; 5 Tanabe Preservative Dentistry, 2-12-18 Mizutani, Higashi-ku, Fukuoka 813-0041, Japan

**Keywords:** older, self-care, *Lactobacillus salivarius*

## Abstract

Objective: A randomized, double-blind, placebo-controlled crossover comparative study was conducted in a healthy older population to assess the usefulness of *Lactobacillus salivarius* WB21 (WB21) ingestion for oral self-care. Methods: The study population included 33 healthy older individuals who were randomly divided into two groups (A and B). Group A consumed WB21 tablets during the first two months and placebo tablets during the following two months. Group B consumed placebo tablets during the first two months and WB21 tablets during the following two months. Before and after ingestion, oral examination, mouth odor test, and saliva collection were performed a total of four times. In addition, health conditions were obtained from a questionnaire survey at the study’s midpoint. Results: Two people in group A and one person in group B dropped out of the study. Thus, 15 people in group A and 15 people in group B were included in the analysis. Over two months of WB21 ingestion, salivary secretory IgA increased significantly (*p* = 0.047) and tongue coating score decreased significantly (*p* = 0.013). The plaque index, bleeding on probing, and mouth odor levels (H_2_S and CH_3_SH concentrations) did not change. During the 6-month study period, no caries, deterioration of periodontitis, or changes in oral health or systemic subjective symptoms were observed. Conclusion: Continuous ingestion of WB21-containing tablets may promote self-care of the teeth and mouths of healthy older adults. Trial registration: R000028335 (UMIN-CTR).

## 1. Introduction

Probiotics are defined as “microorganisms and their growth promoters that can improve the bacterial flora in the gastrointestinal tract and have beneficial effects on the host [1]”. When a microorganism with probiotic function is ingested, it acts on the bacterial flora in the digestive tract to prevent or improve disease while improving the health of the host. In addition, probiotics have an immunostimulatory effect [2] and are involved in suppressing the onset of infectious diseases, cancer, allergies, and other health conditions [3,4,5]. Lactic acid bacteria and bifidobacteria are typical probiotics. Lactic acid bacteria are present in a wide range of environments, from agricultural products and processed foods to the bodies of humans and other animals, whereas bifidobacteria live only in the intestinal tracts of animals. Such bacteria are mild in action and have few side effects, so they are often compared to beneficial insects in agriculture and are attracting attention for preventing and treating disease as replacements for antibacterial drugs and vaccines.

There has been increasing interest in the application of probiotics to improve the oral environment. More than 700 types of microorganism coexist in the human oral cavity, numbering 100 million per 1 mg plaque. Periodontitis and oral malodor develop and worsen with an imbalance of bacterial flora (dysbiosis) [6]. In addition, gingival inflammation and periodontitis may worsen with weakened immunity [7]. Probiotics that normalize the bacterial flora and activate the immune system are expected to be useful for the prevention of such conditions.

*Lactobacillus salivarius* strain WB21 is an acid-tolerant lactobacillus derived from *L. salivarius* WB1004 [8], which is a potentially effective probiotic against *Helicobacter pylori*. We have clinically evaluated the beneficial effects of continuous intake of tablets and oils containing *L. salivarius* WB21 (WB21) on periodontitis, dental caries, and oral malodor [9,10,11,12]. While previous studies have been performed in hospital outpatients, this study focused on maintaining good health and preventing illness in healthy people. As oral health has a great influence on the health of the whole body, it is important to maintain and improve the health of the oral cavity on a daily basis to extend healthy life expectancy. To this end, we investigated the role of continuous intake of lactic acid bacteria in maintaining the health of the oral environment in healthy older adults.

## 2. Materials and Methods

### 2.1. Study Population

Permission for the study was obtained from the Ethics Committee for Clinical Research for Fukuoka Gakuen (approval number 310). The study population consisted of 33 healthy older adult volunteers (19 men, 14 women, average age 70.6 ± 3.7 years) belonging to the Chikushi Minami Senior Club in Chikushino City, Fukuoka Prefecture. The sample size was determined based on a previous crossover study using WB21 tablets [12]. The purpose, significance, method, and duration of the study were fully explained, and all subjects provided written informed consent to participate in the study with sufficient understanding. Those with no teeth, undergoing dental treatment (excluding maintenance), who had taken antibiotics within the past 3 months, who were smokers, and who had dairy allergies, serious metabolic disorders (diabetes, renal disease, liver disease), or malignant tumors were excluded.

### 2.2. Study Design

**Figure 1:**
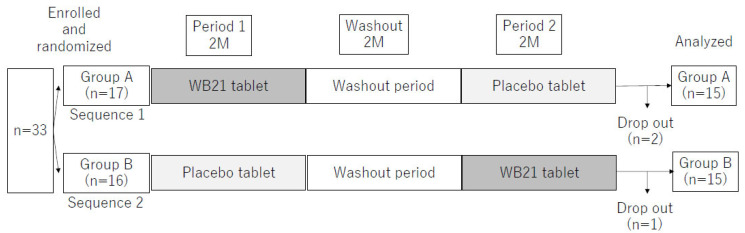
Flowchart of this randomized, double-blind, placebo-controlled crossover comparative study. Both the intervention period and the washout period were 2M (2 months).

This was a randomized, double-blind, placebo-controlled crossover study. The substitution block method was used for random allocation, and deblinding was performed at the time of data analysis. The names of study subjects were listed in the order of the Japanese syllabary, and then numbered from 1. A serial number was assigned to the bags containing the tablets, and the study subjects were given bags with the same number. A credible third party (Hisashi Anan, a professor at Fukuoka Dental College), who was not involved in this study, was in charge of random allocation, bag preparation, and confidentiality of the allocation until data analysis. During the intervention period, the study subjects and researchers were blind to the allocation. The washout period was 2 months, and the study subjects took the tablets containing WB21 or placebo tablets for 2 months each. Oral examination, measurement of the compounds of oral malodor, and saliva collection were performed before and after each period of ingestion of both types of tablet (Figure 1). In addition, at the midpoint of each period, a questionnaire survey and an oral examination were conducted to confirm the intake status and health status. To eliminate the effects of eating and drinking immediately before and cleaning the oral cavity, the subjects fasted and refrained from oral cleaning from the time of waking up in the day of the examination, and all examinations were conducted in the morning at the Chikushi Minami Community Center.

### 2.3. Ingestion Method

The participants took three lactic acid bacteria-containing tablets daily, for a total of 2.0 × 10^9^ cell forming units (cfu) of *L. salivarius* WB21 and 840 mg of xylitol (Minna no Zendamakin WB21 tablet; Wakamoto Pharmaceutical Co., Ltd., Tokyo, Japan). Placebo tablets contained only xylitol and had the same taste, texture, appearance, and shape as the test tablets. The subjects were instructed to place one tablet in the oral cavity after eating three times a day (after brushing) until the tablet dissolved. Considering the adverse effects of highly antibacterial dentifrices and mouthwashes on lactic acid bacteria, during the test period, the participants used standard dentifrice that did not contain antibacterial ingredients (Clinica Lion Mild Mint; Lion, Tokyo, Japan).

### 2.4. Oral Examination

In the oral examination, the number of teeth, plaque Index [13], periodontal pocket depth (PPD) and bleeding on probing (BOP) [14], and tongue coating adhesion status (TCS) determined using the Winkel index [15] were investigated.

### 2.5. Oral Malodor Assessment

To evaluate oral malodor, Oral Chroma^®^ (Nissha FIS, Osaka, Japan) was used to measure the concentrations of three volatile sulfur compounds (hydrogen sulfide (H_2_S), methyl mercaptan (CH_3_SH), and dimethyl sulfide), which are typical oral malodor components. The total concentration of H_2_S and CH_3_SH, which are the main odor components derived from the oral cavity, was used as the outcome index.

### 2.6. Quantification of Salivary Secretory IgA

To quantify secretory IgA (sIgA) in saliva, 1 mL of resting saliva was collected using a saliva collection kit (Saliva Collection Aid; Salimetrics, Carlsbad, CA, USA) on the day of the test. Saliva samples were stored at −20 °C until used for analysis. After all sampling was completed, sIgA in saliva was quantified using an enzyme immunoassay (EIA) with the Salivary Secretory IgA EIA Kit (Salimetrics).

### 2.7. Outcome Index

The outcome indicators were sIgA in saliva, tongue coating adhesion, plaque index, BOP, and oral malodor level (H_2_S + CH_3_SH).

### 2.8. Statistical Analysis

The baseline clinical characteristics of group A (tablets in the order WB21–placebo) and group B (tablets in the order placebo–WB21) were compared using the Mann–Whitney *U* test and the χ^2^ test. The Wilcoxon signed-rank test was used for intragroup comparisons of the 2-month changes in outcome indicators during the WB21 and placebo intake periods. In the intergroup comparison, the amount of change within the group was evaluated using the Mann–Whitney *U* test. With regard to outcome indicators, the carryover effect was calculated for each group (period 1 + period 2), and the *t* test was used to compare groups A and B [16]. The treatment effect was calculated for each (period 1 − period 2)/2, and the *t* test was used to compare groups A and B [16]. The period effect was calculated as (period 1 − period 2)/2 in group A and (period 2 − period 1)/2 in group B, and the *t* test was used to compare groups A and B [16]. SPSS Statistics (Version 22.0; SPSS Japan, Tokyo, Japan) was used for statistical analysis. In all analyses, *p* < 0.05 was taken to indicate statistical significance.

## 3. Results

### 3.1. Baseline Characteristics of the Participants

Three of the subjects (two in group A and one in group B) dropped out of the study due to personal health problems (one man in group A) and family circumstances (two women—one in group A and one in group B), and 30 subjects were ultimately included in the analysis (15 in group A and 15 in group B). The intervention and follow-up were conducted from October 2016 to June 2017. Table 1 shows a comparison of clinical parameters at baseline between groups A and B. There were no differences between the two groups in age, sex ratio, number of teeth, Decayed, Missing, and Filled Teeth (DMFT) index, oral malodor level (H_2_S + CH_3_SH), plaque index, BOP, PPD, TCS, and salivary sIgA concentration.

### 3.2. Analysis of Outcome Index

Table 2 shows the baseline and outcome index values for each of the WB21 and placebo intake periods. Salivary sIgA increased before and after the intervention in both intake periods. Other indicators, such as TCS, plaque index, BOP, and oral malodor levels, decreased. There was a significant increase in salivary sIgA (*p* = 0.047) and a significant decrease in TCS (*p* = 0.013) during the WB21 intake period, but there were no significant differences between the WB21 intake period and the placebo intake period.

**Figure 2:**
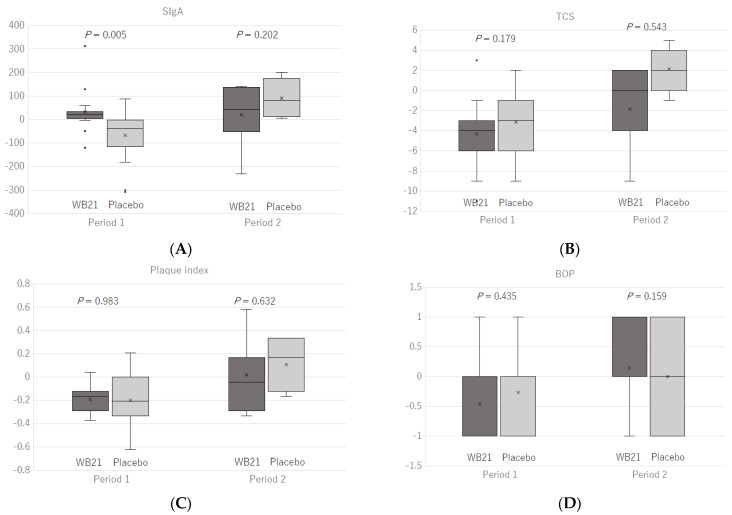
Change in outcome index values during the intervention period. Salivary secretory IgA (sIgA) (**A**), tongue coating score (TCS) (**B**), plaque index (**C**), and bleeding on probing (BOP) (**D**).

A carryover effect was observed in salivary sIgA, and a period effect was observed in salivary sIgA, TCS, plaque index, and BOP (*p* < 0.05). Figure 2A shows the change in salivary sIgA before and after the intervention according to period. In the first half (period taking placebo tablets, and the difference between the two groups was significant (*p* = 0.005). In the second half (period 2), however, it increased in both groups and no significant differences between the groups were observed. TCS, plaque index, and BOP decreased in both groups in the first half (period 1), but the decrease was small overall in the second half (period 2), and TCS and plaque index increased in group A taking placebo tablets (Figure 2B–D).

### 3.3. Occurrence of Adverse Events

Table 3 shows the results of a questionnaire survey conducted at the midpoint of each intake period. Regarding diarrhea, 14.3% answered that it was worse during the placebo intake period, but none answered that it was worse during the WB21 intake period.

## 4. Discussion

In previous clinical studies, the effects of lactic acid bacteria on fever associated with norovirus gastroenteritis [17] and the prevalence of oral candidiasis [18] were investigated in older patients requiring nursing care and frail patients in facilities for older adults. However, there have been no studies regarding maintenance or improvement of oral health in healthy older adults. As the body ages and its physiological function declines with aging, even healthy older adults tend to have poor plaque control due to a decrease in self-care ability. In addition, stomatitis and periodontitis are more likely to occur due to decreased saliva secretion and weakened immunity. As ingestion of *L. salivarius* WB21 leads to an increase in saliva secretion and improvement of periodontitis [10,11,12], it may be useful for coping with the difficulty of oral health management in healthy older adults. Therefore, this study was performed in a population of healthy older adults with an average age of 70.6 ± 3.7 years. In addition, many microorganisms used as probiotics have an immunostimulatory effect [19,20], and so we also examined the effects of continuous ingestion of WB21 on sIgA in saliva.

The results indicted a decrease in tongue coating and a significant increase in sIgA in saliva during the WB21 intake period. The decrease in tongue coating is consistent with the result of previous study [12]. That previous study also found an increased salivary flow rate and reduced numbers of ubiquitous bacteria and *Fusobacterium nucleatum* during the probiotic period [12]. The decreased tongue coating during the probiotic period might be due to improvement in the self-cleaning action of saliva and normalization of the oral bacterial flora. There is a clear relationship between pneumonia in the older population and oral hygiene [21], and it is important to reduce the amount of debris on the tongue. sIgA in saliva is the main mediator of “mucosal immunity” that prevents the invasion of pathogens. Previous studies using *Lactobacillus reuteri* in a young healthy population found no changes in salivary sIgA [22]. However, further research found that individuals with *L. reuteri* in their saliva had significantly higher concentrations of salivary sIgA at the termination of probiotic intake compared with individuals lacking *L. reuteri* [23]. A randomized controlled trial of infants reported increases in salivary sIgA and average daily stool count with continued intake of probiotics [20]. In this study, in a questionnaire survey to investigate the occurrence of adverse events, diarrhea worsened by 14.3% and constipation worsened by 7.7% during the placebo intake period, whereas neither worsened during the WB21 intake period (Table 3). These results suggest that continuous ingestion of tablets containing WB21 contributes not only to oral health, but also to maintenance of general health, such as improvement of the intestinal environment.

On the other hand, in this study, a nonsignificant increase in salivary sIgA was observed even during the placebo intake period, and there were no significant differences in the degree of change between the groups. We observed a carryover effect in salivary sIgA. In previous crossover studies of patients complaining of oral malodor, a 2-week intervention period and a 2-week washout period were appropriate [12]. The intervention period in this study was 2 months, which is 4 times longer than in the previous study, and thus, the 2-month washout period may have insufficient to reduce the effect of WB21. In the results of the first half (period 1) alone, a large difference was observed between the group taking WB21 (group A) and that taking the placebo (group B) (*p* = 0.005) (Figure 2A).

A period effect was observed for sIgA, TCS, plaque index, and BOP. TCS, plaque index, and BOP decreased in both groups in the first half, but the decrease was small overall in the second half, and TCS and plaque index increased in group A while taking the placebo in the second half of the study. Although we predicted a decline in compliance in the second half because of the long period of the intervention, the tablet intake rate was high in both periods and compliance was maintained: 94.7% (WB21) and 91.3% (placebo) in Group A and 87.9% (placebo) and 89.5% (WB21) in Group B. However, it is possible that the attention to oral cleaning habits had diminished in the latter half of the intervention period. In addition, as the second half of the clinical trial was conducted from winter to spring, it may have been influenced by seasonal factors, such as the presence or absence of changes in temperature and humidity, year-end and new-year events, and wearing of face masks in the cold/flu season. Crossover trials are suitable for a small number of subjects and have the advantage of reducing the effects of individual differences. However, future crossover trials should take into consideration the lifestyle and psychological factors of the participants and the season for determining the duration of the intervention and washout period.

The main effects of probiotics are considered to include improvement of the resident flora from dysbiosis to symbiosis and activation of the host’s immune system. In previous studies, continuous intake of *L. salivarius* WB21 for 2 weeks significantly decreased total bacterial numbers, while no change in the composition of the bacterial flora in the oral cavity was observed [11]. The application of *L. reuteri* to mucositis and peri-implantitis led to significant changes in the abundances of several bacterial species, but this effect was limited [24]. Thus, the effects of probiotics on the flora of the oral cavity appear to be mild. In relation to the immunostimulatory effect, salivary sIgA levels were significantly elevated during the intervention period in this study, and a particularly large difference was observed in the first half (period 1). On the other hand, the effect was not observed during the second half (period 2) due to the carryover effect and period effect. A previous study reported that salivary sIgA levels were affected by the presence of *L. reuteri* in saliva after ingestion [23]. The effect of probiotics on salivary sIgA may be affected by host reactivity and lifestyle changes. Based on the results presented in Table 3, the study subjects did not feel that the probiotic tablets were associated with subjective symptoms, either good or bad. Overall, the mildness of the effects might make it difficult to demonstrate the efficacy of probiotics. On the other hand, probiotic bacteria work to improve the overall condition of the host through additive small changes in multiple contexts.

There is abundant evidence that polyphenols have beneficial effects on human health by altering the gut microbiota, similar to probiotics [25]. In addition, light-activated antibacterial therapy is a new method of locally suppressing target bacteria [26]. Achieving additive or synergistic effects by combining these methods, which have no harmful or side effects, is also possible, and research in this area will become increasingly important in the post-antibiotic era.

## 5. Conclusions

When the lactic acid bacterium *L. salivarius* WB21 was continuously ingested for 2 months in a healthy older population, a decrease in tongue coating and an increase in salivary sIgA were observed. These observations suggest that WB21 may be useful for maintaining oral health in the healthy older population.

## Figures and Tables

**Table 1:** Baseline clinical parameters for group A subjects (WB21→placebo; *n* = 15) and group B subjects (placebo→WB21; *n* = 15). Values represent the number of participants or median (interquartile, IQR).

Parameter	15 Allocated to Sequence 1 (WB21–Placebo)	15 Allocated to Sequence 2 (Placebo–WB21)	*p*-Value
Age (years)	70.0 (68.0, 71.5)	71.0 (68.0, 73.0)	0.367
Female/Male	6/9	6/9	1.000
Number of teeth	28.0 (26.0, 29.0)	27.0 (25.0, 29.0)	0.713
DMFT	17.0 (14.0, 19.5)	15.0 (13.0, 16.5)	0.174
H_2_S & CH_3_SH (ng/10 mL)	1.7 (0.7, 6.1)	2.0 (0.9, 10.7)	0.713
Plaque Index	0.1 (0.0, 0.1)	0.0 (0.0, 0.1)	0.174
BOP(−)/BOP(+)	10/5	10/5	1.000
PPD (code 0, 1, 2)	1.0 (0.5, 1.0)	1.0 (0.0, 1.0)	0.325
TCS	0.0 (0.0, 2.0)	1.0 (0.0, 2.5)	0.567
sIgA (µg/mL)	177.1 (131.9, 308.1)	152.5 (108.6, 224.1)	0.436

Female/Male and BOP(−)/BOP(+): chi-square test. All other analyses used the Mann–Whitney *U* test.

**Table 2:** Change in clinical parameters in the WB21 treatment period (*n* = 30) and the placebo period (*n* = 30).

Parameter	WB21 Period		Placebo Period	
	BL	2 M	BL	2 M
sIgA (µg/mL)	179.2 (116.5, 295.2) *	207.2 (142.5, 296.0) *	171.9 (109.0, 252.7)	194.4 (117.5, 265.6)
TCS	4.0 (2.0, 5.0) *	2.0 (0.0, 3.0) *	3.0 (2.0, 4.8)	2.0 (1.0, 4.8)
Plaque Index	0.2 (0.1, 0.3)	0.1 (0.0, 0.2)	0.2 (0.0, 0.4)	0.1 (0.0, 0.2)
BOP	0.5 (0.0, 1.0)	0.0 (0.0, 1.0)	1.0 (0.0, 1.0)	0.0 (0.0, 1.0)
H_2_S & CH_3_SH (ng/10 mL)	3.5 (1.3, 13.6)	1.1 (0.6, 6.4)	4.3 (0.9, 7.5)	1.5 (0.5, 6.2)

* *p* < 0.05 between BL (baseline) and 2 M (2 months) by the Wilcoxon signed-rank test.

**Table 3:** Subjective symptoms associated with tablet intake.

Question	Period (Number of Responses)	Deterioration % (Number of Responses)	No Change % (Number of Responses)	Improvement % (Number of Responses)
Gastrointestinal symptoms				
Diarrhea	WB21 (28) Placebo (28)	0.0 (0) 14.3 (4)	96.4 (27) 85.7 (24)	3.6 (1) 0.0 (0)
Constipation	WB21 (27) Placebo (26)	0.0 (0) 7.7 (2)	92.6 (25) 88.5 (23)	7.4 (2) 3.8 (1)
Physical symptoms				
Fatigue	WB21 (30) Placebo (28)	0.0 (0) 3.6 (1)	100.0 (30) 96.4 (27)	0.0 (0) 0.0 (0)
Appetite	WB21 (30) Placebo (29)	0.0 (0) 0.0 (0)	100.0 (30) 100.0 (29)	0.0 (0) 0.0 (0)
Awakening	WB21 (30) Placebo (29)	0.0 (0) 0.0 (0)	100.0 (30) 100.0 (29)	0.0 (0) 0.0 (0)
Sleep quality	WB21 (30) Placebo (29)	0.0 (0) 0.0 (0)	100.0 (30) 96.6 (28)	0.0 (0) 3.4 (1)
Oral cavity symptoms				
Tooth pain	WB21 (30) Placebo (29)	3.3 (1) 3.4 (1)	96.7 (29) 96.6 (28)	0.0 (0) 0.0 (0)
Gingival pain	WB21 (29) Placebo (29)	3.4 (1) 3.4 (1)	96.6 (28) 96.6 (28)	0.0 (0) 0.0 (0)
Swelling in the gums	WB21 (30) Placebo (29)	3.3 (1) 3.4 (1)	96.7 (29) 96.6 (28)	0.0 (0) 0.0 (0)
Discomfort in the mouth	WB21 (30) Placebo (29)	6.7 (2) 6.7 (2)	86.7 (26) 86.2 (25)	6.7 (2) 6.7 (2)
Bad breath	WB21 (30) Placebo (29)	0.0 (0) 0.0 (0)	96.7 (29) 96.6 (28)	3.3 (1) 3.4 (1)

## Data Availability

The data presented in this study are available upon request from the corresponding author. The data are not publicly available due to restrictions, e.g., privacy or ethical concerns.
